# Opening Pandora’s box in the emergency room: a secondary analysis of existential needs from observational, registered nurse, and patient perspectives

**DOI:** 10.1186/s12912-026-04601-3

**Published:** 2026-03-30

**Authors:** Veronica Pavedahl, Martina Summer Meranius, Åsa Muntlin

**Affiliations:** 1 Department of Health Sciences, Faculty of Health, Science, and Technology, Karlstad University, Karlstad, Sweden; 2 Center for Clinical Research and Education, Region Värmland, Karlstad, Sweden; 3 School of Health, Care and Social Welfare, Mälardalen University, Eskilstuna Västerås, Sweden; 4 Department of Medical Sciences, Uppsala University, Uppsala, Sweden; 5 Department of Ambulance and Emergency Care, Uppsala University Hospital, Uppsala, Sweden

**Keywords:** Existential needs, Nurse-patient relations, Emergency room, Emergency nursing, Fundamental care, Person-centered care, Secondary analysis, Qualitative research

## Abstract

**Background:**

Emergency rooms prioritize rapid, life-saving interventions, often at the expense of patients’ psychosocial and existential needs. Patients in these settings are particularly vulnerable, yet such needs are frequently overlooked. Despite growing emphasis on person-centered care, knowledge of how existential needs (e.g. fear, anxiety, vulnerability) are expressed and addressed in emergency rooms remains limited. This study aims to describe how existential needs are expressed in the emergency room context.

**Methods:**

A qualitative secondary analysis was conducted using 108 field notes, 14 registered nurse interviews, and 15 patient interviews viewing emergency room care through a Fundamentals of Care framework lens. An inductive approach was inspired by key existential concepts described in a review and relevant to acute care. Reflexive thematic analysis was applied to each dataset, followed by cross-data comparison to identify patterns of existential needs.

**Results:**

The secondary analysis resulted in four themes; *Existential vulnerability in a technical environment*,* Ensuring trust through presence and communication*,* Violations of privacy and dignity*,* and Moments of meaning-making through connection* showing that existential needs in the emergency room are widespread and closely connected to both patients’ experiences and registered nurses’ care practices. Patients faced sudden transitions, fear, and emotional vulnerability, while registered nurses balanced these needs with organizational constraints. Existential concerns were expressed verbally and non-verbally and shaped by the environment, communication, and presence. Even brief, attentive encounters were experienced as meaningful and created a sense of safety in the stressful clinical setting.

**Conclusions:**

Addressing existential needs is integral to emergency care. Recognition and responsive care can mitigate patients’ emotional isolation and enhance safety, despite organizational constraints, highlighting the importance of integrating existential support with medical treatment. The organization should enable healthcare professionals to recognize and respond to existential needs as an integrated part of patient assessment and care interventions, supporting a more person-centered and holistic care in acute environments. This study highlights the importance of addressing existential needs within the emergency room, for both patients and registered nurses. Organizational constraints and ethical tensions challenge person-centered fundamental care, while underscoring safety as a crucial condition for meaningful and compassionate encounters. The findings offer guidance for emergency care professionals on integrating existential and relational awareness into practice, education, and policy.

**Trial registration:**

Clinical trial number: Not applicable.

## Background

Emergency departments (EDs) are traditionally oriented towards rapid and precise medical and technical interventions, often described as complex, stressful, and overwhelming environments [1]. The primary focus lies in managing life-threatening conditions using advanced technology and standardized treatment routines [2]. However, within the ED, the emergency room—a designated unit for the care of patients with the most critical and life-threatening conditions—is a particularly intense environment. Here, the emphasis on acute medical care can sometimes overshadow other important aspects of emergency care such as psychosocial and relational aspects [3]. Patients in emergency rooms who experience life-threatening illnesses or injury do express existential needs—yet these are not always assessed or addressed [4].

Becoming a patient in an emergency room might be challenging for several reasons. Patients have described the quick, systematic assessment and treatment as strict and impersonal and say that, combined with an acute health problem, the environment can feel stressful [5]. It is an intense work environment where healthcare professionals face difficult decisions [6]. Due to the time-sensitive and demanding nature of the environment of the emergency room, coupled with a diverse array of patient needs to address, providing optimal and holistic care there can pose challenges [7]. Patients in this context are vulnerable, exposed, and dependent—more so than in many other care settings [8]. This vulnerability can be distressing, and may contribute to unnecessary suffering [9]. Often, this psychosocial dimension—the emotional needs of patients—has been regarded as something outside the immediate scope of emergency care [5, 10]. There is often a lack of awareness of how these needs actually form an integral part of ED care. This tendency to overlook patients’ emotional and psychosocial needs is not confined to emergency care but has also been noted across other clinical contexts [11, 12]. Yet, existential thoughts and emotions often become particularly prominent when an individual or their loved ones are affected by a severe or life-threatening illness [13]. Therefore, within healthcare, the inclusion of this element is considered important for achieving holistic care.

In recent years, however, a growing emphasis on person-centered care has highlighted the importance of adopting a more holistic perspective that integrates relational, emotional, and physical aspects of care [14]. One response to this development has been the formulation of the Fundamentals of Care framework, proposed as a means of structuring and delivering person-centered fundamental care across diverse healthcare settings [12, 15]. This framework outlines three interrelated dimensions: the establishment of a caring relationship with the patient; the assessment and provision of physical, relational, and psychosocial fundamentals of care; and the broader context in which care is delivered. To achieve person-centered care that adequately addresses the patient’s fundamental care needs, all dimensions must be integrated, as none can be omitted without compromising the overall quality of care [12, 15]. Although existential needs are fundamental human needs, they are not explicitly included in the terminology of the Fundamentals of Care framework. This omission is not merely a consensus issue. In the beginning, the framework highlighted dying and end-of-life care, and studies examining things like stroke survivors’ care included emotional reactions under the rubric of dignity [16]. These elements, however, were excluded in the Delphi study [12]—not due to a lack of perceived importance but because divergent terminology and culturally influenced expressions prevented agreement. Thus, existential needs continued to be recognized as significant yet were not formally incorporated into the Fundamentals of Care framework. Even so, subsequent studies have shown that patients themselves articulate these needs when discussing fundamental care [4, 17]. These findings indicate that existential concerns are an integral aspect of patient experiences of care.

### Conceptualizing existential needs

The concept of “existential” encompasses a range of interpretations and connotations, often used in research, albeit with different meanings [18]. In a recent review of 138 articles across Scandinavian healthcare journals, Nygaard, Austad [19] found that while the term frequently appears—often paired with other words such as existential pain, existential struggle, or existential encounter—it is rarely defined and there seems to be no consensus on the concept’s meaning. Despite this inconsistency, Nygaard et al., [19] synthesized a working definition to guide further research. They propose that, in a healthcare context, the existential refers to “the fundamental, basic condition of being a human”. It encompasses the inescapable reality of life and death, uncertainty, and the human drive to seek meaning—particularly in moments of crisis. Existential concerns, they note, often manifest in the tension between suffering and reorientation, and meaning and meaninglessness.

To the best of our understanding, there is limited knowledge of how existential needs are expressed by patients, perceived by healthcare professionals, and integrated into care, particularly in the emergency room context. In this study, existential needs are understood as needs related to how individuals make sense of their situation, confront vulnerability, uncertainty, and mortality, and seek meaning, reassurance, or acknowledgment in situations of existential distress. In relation to fundamental care needs, existential needs foreground how patients interpret and make sense of their situation. By combining multiple perspectives, this study aims to capture existential needs as individual, relational, and organizational phenomena. Combining insights from different perspectives enables a nuanced understanding of how existential needs arise and how they are, or are not, acknowledged and addressed in the emergency room.

### Aim

The aim is to describe how existential needs are expressed in the emergency room context.

## Methodology

### Design

This study employed a qualitative secondary analysis, which leverages existing data to answer new questions or validate findings, offering deeper insights into trends and patterns [20, 21].

The data originated from the first author’s (VP) doctoral project focusing on person-centered fundamental care in the emergency room, structured around the Fundamentals of Care framework [12]. This framework guided the original data collection, providing a conceptual foundation for exploring patients’ and registered nurses’ (RNs’) experiences of fundamental care. The original studies did not focus specifically on existential needs. While such dimensions were noted during the initial analyses, they were not further explored due to the different primary aims. The identification of implicit existential dimensions in the original dataset prompted this secondary analysis, which explores how existential needs are expressed and addressed in emergency room settings.

### Data sources

The dataset consisted of material originally collected, in Sweden, between 2019 and 2024 as part of the aforementioned doctoral project. Because the project’s conclusions directly informed the formulation of new research questions, the existing data were considered highly relevant for the present study. It includes 108 field notes, 14 interviews with RNs, and 15 interviews with patients, thereby capturing multiple perspectives on the care provided in the emergency room. Field notes were collected through observations, documented using an observation protocol based on the Fundamentals of Care framework [12]. Documented in the field notes were the observer’s own reflections on and thoughts about the event as well as notes about the setting, the activities that occurred, the atmosphere in the emergency room, the environment (furnishings, smells, sounds), and interactions among the team. To access the participants’ experiences, the interviews were conducted using open-ended questions. Semi-structured interview guides were used. These guides were developed for two original studies and have been previously published [4, 22]. The present study constitutes a secondary analysis of these interview data. Interviews with RNs were conducted face to face, and those with patients by telephone; telephone contact was used as data were collected during the COVID-19 pandemic. Participants were informed both verbally and in writing about the study’s objectives, the confidentiality of data management, and the voluntary nature of participation; written informed consent was subsequently obtained. For the original studies, inclusion criteria for RNs were current employment in the emergency room, with prior ED experience and completion of mandatory internal training. For patients, inclusion criteria were age 18 or older and the ability to understand and speak Swedish. Patients were excluded if still admitted to in-hospital care, expected to have persistent cognitive failure, had a recent suicide attempt, or were intoxicated, for ethical reasons. RNs were 28–61 years old (mean 40.2), 11 females and 3 males, with 1–14 years of emergency room experience (mean 6.1). Patients were 32–84 years old (mean 65.0), 6 women and 9 men, presenting with a variety of conditions (e.g., heart failure, chest pain, syncope, sepsis) and typically spending 60 min or more in the emergency room before transfer for further care.

The field notes have not been previously published, and while parts of the interview material informed previous analyses, the present study applies a novel focus on existential needs. While the doctoral project examined fundamental care more broadly, the present study specifically reanalyzes the data with a focus on existential needs. Table 1 summarizes key characteristics of each data source, including collection period, units, and original analytic focus.

**Table 1:** Key characteristics of original data

Data source	Period	*N* (units)	Aim
Field notes from observations	May – November 2019	108	To explore how fundamental care needs of life-threateningly ill or injured patients are met in emergency rooms
Interviews with RNs	May – November 2019	14	To explore how RNs in the emergency room describe their work approach and prerequisites for meeting life-threateningly ill patients’ care needs from the perspective of a person-centered fundamental care framework
Interviews with patients	April – May 2022	15	To describe fundamental care needs experienced by patients in the emergency room with a life-threatening condition

### Data analysis

A reflexive thematic analysis (RTA) according to Braun and Clarke [23] was undertaken to identify and interpret patterns of meaning across the datasets. Importantly, the analysis was conducted dataset by dataset (i.e. interview for interview, field note for field note), before moving to cross dataset comparison. This approach allowed each data source to be considered on its own terms, while maintaining openness to the emergence of new patterns and themes. This reflects a scientific and reflective attitude, emphasizing reflexivity and openness to the phenomenon throughout the analytic process.

In Phase 1, all authors familiarized themselves with the dataset through repeated readings of the transcripts, noting initial impressions. In Phase 2, the research team collaboratively produced inductive codes to capture features of the data that were meaningful in relation to the research aim. Coding was discussed iteratively, with differences in interpretation explored through reflective dialogue and resolved through negotiated consensus, highlighting the researchers’ active role in shaping the analytic process. These codes formed the basis for constructing preliminary themes in Phase 3, in which visual mapping techniques were used to explore conceptual relationships and patterns across the data. During the fourth phase the research team collaboratively reviewed, refined, and actively reshaped the themes, paying attention to internal consistency within themes as well as the relationship between them and the entire dataset. Some themes were revised, merged, or discarded through an iterative process of analysis and discussion. Phase 5 involved naming and defining each theme and subtheme, with attention to their distinct analytic focus. In Phase 6, the thematic narrative was crafted and produced, supported by illustrative quotes and interpretive commentary. Throughout the process, reflexivity was actively practiced [23]. The authors’ clinical backgrounds as RNs—two with experience in caring for patients with life-threatening conditions—were acknowledged and the analysis was approached with a reflective attitude. Potential influences of these preunderstandings were discussed within the research team. Interpretive differences were explored through reflective dialogue and resolved through negotiated consensus. The Reflexive Thematic Analysis Reporting Guidelines (RTARG) [24] were applied to ensure methodological congruence in the conduct and reporting of studies using RTA.

**Fig. 1:**
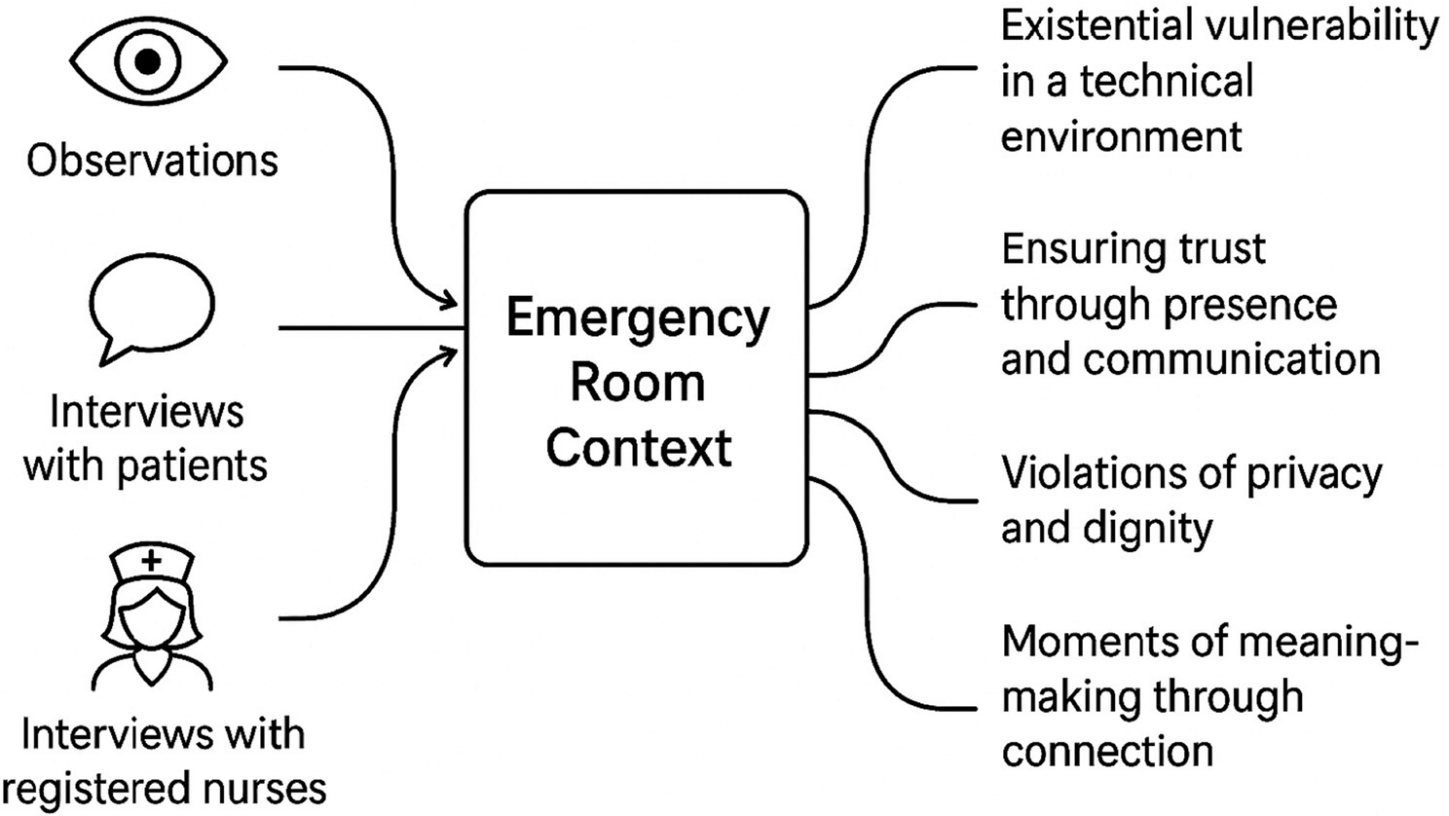
Existential needs in the emergency room

To identify existential needs in our data we drew on key attributes relevant to nursing and emergency care, outlined in a comprehensive literature review by Nygaard et al., [19]; these include concepts such as *anxiety*,* caring encounter*,* crisis*,* death*,* dialogue*,* distress*,* fear*,* future*,* health*,* health at stake*,* holistic care*,* hope*,* hopelessness*,* human needs*,* insecurity*,* isolation*,* loneliness*,* loss*,* meaning*,* meaninglessness*,* pain*,* safety*,* social alienation*,* struggle*,* suffering*,* threat*,* and transition*. Rather than applying them as deductive codes, we used them as a conceptual lens to guide interpretation. This approach allowed us to recognize expressions of existential need while remaining open to new insights in the emergency room context (see Fig. 1).

### Ethical considerations

Approved by the Swedish Ethical Review Authority (Dnr. 2019 − 00506) and conducted in accordance with the Declaration of Helsinki [25]. All collected data adhered to the General Data Protection Regulation [GDPR] following rules and regulations of the University in question.

## Results

This secondary analysis resulted in four themes describing how existential needs are expressed and responded to as experienced by patients, reflected upon by RNs, and observed in emergency room practice.

### Theme 1: Existential vulnerability in a technical environment

The emergency room emerged as a space where existential vulnerability was both exposed and intensified. Descriptions depicted being thrown from everyday life into a state of crisis within the span of just a few minutes. Patients described sudden transitions from health to life-threatening conditions as disorienting and surreal, often accompanied by fear, loss of control, and proximity to death. These experiences were marked by existential questioning—“*Am I going to die?*”—and a sense of being thrust into a liminal state between life and death. The rapid and overwhelming nature of the emergency room can heighten patients’ sense of existential uncertainty:

*Everything happened so fast—one moment you’re healthy and the next there’s a threat to your life*,* with wires and cables all over your body. And with that there come people—there were people everywhere…/ /… No one explained why it (the monitoring equipment) was beeping*,* which was a bit anxiety-provoking. I didn’t know if it meant that I was really ill. The room was quite high-tech. And then suddenly*,* just as quickly as everyone had entered the room*,* they were gone. (Participant P:1)*

RNs acknowledged these expressions of anxiety and distress, particularly in moments marked by fear, pain, and uncertainty about life and death. However, the pace and priorities of the emergency room frequently restricted their ability to fully address these needs. RNs highlighted the constraints of the emergency room environment, which prioritized rapid stabilization over emotional and existential support, leaving little room for existential dialogue. They described a delicate balance of engaging with patients’ deepest fears, aware that exploring such emotions could unleash complexities they might not have the time or resources to manage:

*It’s a bit like choosing whether or not to open Pandora’s box. Sometimes I avoid even asking if the patient is upset because I don’t know what will come out of them. And if something comes up that I don’t have time to handle*,* what do I do then? I have other patients…For me as a registered nurse*,* it comes naturally to take care of something like that—to be present with the patient in that moment*,* to comfort them—but it’s not built into the organizational system to handle questions about life and death*,* even though that’s what we’re hands-on working with (silence). (Participant RN:6)*

Field notes revealed that the emergency room, primarily designed for physical stabilization, also became a setting where both patients and RNs were confronted with existential dimensions—patients with immediate threats to their life, loss of control, and vulnerability, and RNs with the challenge of responding to these needs amid organizational constraints. Existential anxiety was often communicated through nonverbal cues such as crying, silence, or hyperventilation, and responses varied depending on time, staffing, and organizational priorities. In some cases the patient remained alone in their distress, while in others the RNs offered calm reassurance:

*Nurse responds calmly: ‘We’re here to help you.’ (Field note 87)*

### Theme 2: Ensuring trust through presence and communication

A recurring theme across all data sources was the importance of presence and communication in alleviating existential distress. There was a strong desire not to be reduced to a medical case but to be recognized as a whole person—as someone, not something. When decisions were made without explanation or inclusion, patients described feelings of insecurity, loss of agency, and isolation. In contrast, being addressed by name, understood in context, and given space to voice one’s fears and needs fostered a sense of trust and meaning. Patients emphasized how small gestures—eye contact, a hand on one’s shoulder, or calm verbal reassurance—created a sense of safety. Many patients interpreted the mere presence of life around them—staff moving, speaking, attending to others—as a sign of vitality and reassurance:

*The first thing I remember is being placed on a narrow stretcher and being asked a lot of questions. It’s a bit blurry. I remember that they were very kind to me. They stood by my side*,* placed a hand on my shoulder*,* talked to me*,* and watched over me. They were there—I was continuously informed about what was happening*,* and I got answers when I asked questions. I felt safe. I felt safe and I trusted them; they seemed competent because they knew about me and what to do with someone in my condition. (Participant P:5)*

Yet this presence was not always accompanied by engagement. Several patients described moments of isolation, particularly during transitions—such as waiting to be discharged—when attention from the healthcare professionals diminished. These moments of reduced interaction contributed to a sense of abandonment. At the same time a form of self-restraint was evident, whereby patients refrained from voicing their concerns or needs, anticipating that healthcare professionals lacked the time or capacity to respond. Patients reflected on the delicate tension between wanting to speak about their deeper concerns and fearing the potential consequences of doing so, aware that raising sensitive topics could lead to emotional responses that might not be fully addressed in the fast-paced environment:

*You’re left completely on your own; you’re left with questions. It’s also something you might not dare to bring up. I mean*,* you know what the emergency room’s for—it’s about survival. And*,* as a patient*,* you’re not really inclined to mention things that aren’t directly survival-related. I*,* for example*,* wouldn’t talk about that kind of thing in an emergency room. If you start talking*,* you want the chance to finish—and that time just isn’t available in there (silence). (Participant P:15)*

RNs recognized the importance of presence and communication in alleviating patients’ distress and described efforts to tailor their communication to the patients’ personal needs, often strategically using tone, timing, and physical proximity to foster trust. Illustrating this, an RN stated:

*Usually*,* when you talk to the patient they tend to become calmer*,* more at ease and settled—if they’ve received the help they needed. But someone who doesn’t feel seen or acknowledged*,* and hasn’t gotten*,* say*,* pain relief*,* will keep calling out for help in different ways. (Participant RN:4)*

Field observations confirmed that communication often served as a tool for reassurance, even when verbal content was minimal. The emergency room environment itself played a dual role in shaping the experience of trust and safety. Alarms, overlapping conversations, and rapid movement could evoke confusion and fear. However, despite time pressures, moments of genuine presence and attuned communication were noted as deeply meaningful.

### Theme 3: Violations of privacy and dignity

The emergency room emerged as a setting where dignity was particularly fragile. Limited privacy and proximity to others’ suffering or death left patients exposed to conversations and events they could not avoid. While some patients acknowledged that staff attempted to provide screening or shielding, these efforts were not always sufficient. Within this environment they risked being reduced to passive recipients of care, unable to shield themselves from what unfolded around them, intensifying experiences of vulnerability. One patient reflected:

*Then I needed to pee as well*,* but there were other patients in the same room. Screens were put up between me and the person next to me*,* but it still felt strange to urinate in that situation. And you hear everything. You hear the healthcare professionals talking to each other and other patients*,* you hear other patients’ social security numbers and what they’ve been through. You don’t see anyone*,* but you hear them. Some of the things I heard I wish I hadn’t. (Participant P:9)*

RNs described how organizational constraints often forced them to prioritize efficiency over relational presence. Decisions and prognoses were sometimes communicated openly, with patients treated in close proximity and within earshot of conversations about treatment futility. Such practices risked violating patients’ privacy and dignity and exposing bystanders to distressing experiences of death and suffering, while RNs struggled to mitigate these effects within existing limitations. One RN described how a patient had overheard a cardiac arrest announcement and witnessed that patient’s subsequent death, while lying unacknowledged nearby:

*So*,* you know*,* someone calls out loudly*,* ‘Cardiac arrest arriving in one minute*,*’ so he knew. Then the cardiac arrest case came in*,* it was chaotic*,* and the patient died. And I just thought*,* ‘Oh God*,* that poor person lying next to all this.’ He wasn’t used to seeing someone die like we are. So*,* I just went over and asked how he was doing*,* and he just said*,* in a scared voice*,* ‘No*,* I’m fine*,* you just keep working.’ It felt like he didn’t dare to say more. But really—what can you say*,* lying there while someone dies right next to you? (Participant RN:7)*

The environment was described as alienating and sometimes profoundly undignified, particularly for dying patients, who were not always afforded the discretion and space they deserved. The lack of privacy, communication, and attentiveness could exacerbate patients’ vulnerability and compromise the provision of a dignified death:

*It was the communication around him that was really terrible. Someone actually said outright*,* ‘So*,* what exactly are we supposed to save here?’ The last thing he heard was just people discussing how little they were going to do. That was truly undignified for this patient. In the end*,* the patient died*,* still almost completely covered in feces*,* and (silence)… and yes*,* when those words are said*,* ‘We’re not doing anything more now*,*’ it feels like many also emotionally let go of the patient. But there was still so much left to do to make sure he had a dignified death (silence). (Participant RN:9)*

Field notes illustrated how unclear roles, overcrowding, and the lack of spatial boundaries in the emergency room risked undermining patients’ privacy and dignity. In contrast, clear task distribution and structured collaboration contributed to a sense of calm, even under acute conditions:

*Fifteen people in the room*,* yet it remains calm and quiet. The healthcare professionals speak softly and calmly; assessments and interventions are carried out at a rapid pace without giving the impression that it’s a highly acute situation. Continuous information is provided*,* the surroundings are screened off*,* and relatives of the other patient are moved out. (Field note 23)*

### Theme 4: Moments of meaning-making through connection

Despite the emergency room’s clinical focus and structural limitations, patients described moments when their existential needs were met—brief instances of connection, recognition, and hope that anchored them in their humanity. These encounters, though often fleeting, held profound meaning and served as existential touchpoints in an otherwise disorienting environment. Patients recalled how simple gestures—someone standing beside them, making eye contact, or offering words of encouragement—were recalled as transformative, creating a sense of safety and coherence amid uncertainty. They described how interactions with healthcare providers, especially those marked by empathy and recognition, offered moments of hope and coherence:

*Well*,* what I really appreciated was when someone came and sat next to me and just talked. Even though I was so ill*,* I could tell they were trying to cheer me up—but also to see if I was confused. I told them I wanted to live at least another six months*,* so I’d have time to meet these two new grandchildren. And they were just wonderful*,* because both of them—independently—gave me the same response. They said*,* ‘Mm-hmm*,* we’ll make sure that happens’ (laughs). I mean*,* they were just so nice to me. I’d say they were professional. I was lying there*,* amazed at how much attention I was getting. (Participant P:12)*

RNs reflected on the importance of being emotionally present and responsive, even when time was limited. Presence, in this context, was understood not as a matter of time spent but as an intentional stance—an ethical responsiveness expressed through attention, tone, and embodied calm. They described how finding a small window of opportunity to offer recognition, care, or clarity could restore coherence and dignity, fostering a sense of safety, especially when patients were vulnerable, confused, or in distress:

*A good example is patients who are intoxicated. Sometimes I write a note and send it with a friend who seems a bit more sober—something like*,* ‘You need to see a dentist*,* because when you wake up tomorrow you won’t like what you see.’ And I know there’s a chance that information won’t land at all [if I’m the one communicating it]. Some patients are in shock or too overwhelmed to take in what we’re saying. But they don’t get that information in the emergency room—it’s up to them if they have someone to reach out to. (Participant RN:1)*

Field notes captured instances in which brief but intentional interactions transformed the clinical encounter into a space of existential support, even amidst the emergency room’s fast pace, fragmented communication, and environmental stressors. This was observed as RNs frequently responded to patients’ expressions of fear, distress, and questions about life and death with calm reassurance, attentive presence, and small gestures that acknowledged the patients’ vulnerability and humanity:

*Two patients in the emergency room. The RN sits briefly with one of them*,* listens*,* asks questions*,* nods*,* smiles. The phone rings in the RN’s pocket. Without answering or even looking at it*,* the RN hands it to a colleague. (Field note 40)*

## Discussion

This study highlights a striking paradox in emergency room care: While the environment is designed to preserve life through rapid intervention, the very logic of medical urgency can inadvertently suppress existential care. Although the empirical material derives from previous studies, this secondary analysis focuses explicitly on existential needs from three perspectives (RNs, patients, and field notes), generating new insights not further explored in the original analyses. This study contributes by showing how existential needs are often indirectly expressed or deliberately withheld by patients, shaped by the organizational conditions of emergency care, including time pressure and lack of privacy, and negotiated across patients, RNs, and the care environment, rather than residing solely within the individual. Yet, as the findings show, existential concerns are not peripheral but central to the patient’s experience of emergency care—emerging immediately upon arrival and shaping how the situation is perceived, endured, and remembered. This paradox reflects a deeper organizational and cultural logic that privileges technical efficiency over relational presence, suggesting that the neglect of existential needs is not an individual shortcoming but a systemic characteristic of contemporary emergency care.

The findings reveal that existential needs arise the moment a person enters the emergency room. These experiences are often intensified by the abrupt transition from everyday life to a life-threatening situation. Patients’ descriptions of being “thrown” into crisis align with Nygaard et al., [19], who argue that existential concerns in acute care are contextually rooted yet rarely acknowledged within clinical routines. Our findings show how the emergency room acts as a crucible for these concerns, rather than simply a setting for technical intervention.

Moreover, patients withheld existential questions or emotional expressions, anticipating that staff lacked the time or capacity to respond, consistent with Lindström et al., [26]. RNs, on the other hand, described a hesitation to engage in existential dialogue for fear of “opening Pandora’s box” without sufficient time or resources to address what might surface. This dynamic reveals how structural and systemic constraints risk leaving central aspects of the patient’s experience unaddressed, effectively rendering their existential needs invisible within routine clinical practice [27]. However, existential support and acute medical care are not mutually exclusive. Rather, the findings suggest that they coexist even in the most time-pressured situations, opening opportunities to integrate existential care as an essential part of person-centered fundamental care without compromising medical priorities.

A key insight from this study is the ethical tension experienced by RNs as they navigate the boundaries between life-saving urgency and existential responsiveness. While prioritizing physical stabilization is clinically necessary, neglecting existential needs risks compromising the moral and relational foundation of nursing itself. In line with Gamst-Jensen et al., [3], our results indicate that ethical considerations and responsibility, in this sense, should not be limited to end-of-life care but should instead be integrated into everyday emergency practice. Attending to patients’ existential needs is not a distraction from medical urgency but rather an affirmation of professional and moral integrity.

Furthermore, the findings reveal a mismatch between patients’ expressions of existential needs and the RNs’ responses. Whereas patients articulated fear, vulnerability, or questions about meaning, RNs responded indirectly—through communication, reassurance, or physical presence—rather than with direct existential dialogue. Such dialogue might involve, for instance, briefly acknowledging the patient’s emotional expression and encouraging further reflection. Although seemingly minor, these moments may offer opportunities to attune the care to patients’ immediate existential concerns. While indirect responses might provide comfort, they may fall short in fully addressing patients’ deeper concerns. This highlights a tension between ethical intention and the constraints in the emergency room environment: RNs recognize patients’ existential needs but are constrained by organizational routines and the potential complexity of the patients’ responses. While these challenges appear to be particularly pronounced in emergency care, they are not unique to this setting. Nevertheless, their difficulty should not be taken as justification for overlooking patients’ existential needs or accepting that contextual barriers make such engagement unattainable. This resonates with Morse [28] argument that alleviating suffering requires recognition and a compassionate, responsive presence. Enabling more explicit existential dialogue would likely require supportive structural and cultural conditions—such as legitimizing brief but meaningful conversations, providing training in existential communication, and fostering a climate in which acknowledging patients’ emotional and existential expressions is considered an integral component of care. Such conditions align closely with principles of person-centered care. Furthermore, our findings resonate with literature on moral distress among RNs in emergency care, showing that professionals experience discomfort when systemic constraints prevent them from providing holistic care in alignment with their values [29, 30]. In the longer run, separating psychosocial care needs from physical care needs might negatively impact the delivery of a person-centered fundamental care, which may manifest as missed or delayed nursing care [31]. Duhalde et al., [32] encourage healthcare professional dialogues regarding workplace culture and emergency nursing practices in relation to nursing responsibilities and missed nursing care.

Our findings illustrate how patients may be exposed to distressing events, overhear sensitive information, or feel reduced to passive recipients of care. The emergency room environment itself often compromises patients’ privacy and challenges their sense of dignity [33], and organizational and environmental constraints—such as shared spaces, open communication, overcrowding, and a lack of physical barriers—intensify their existential vulnerability [34]. These experiences can undermine patients’ sense of self and exacerbate feelings of alienation. Research supports these observations, emphasizing that maintaining patient dignity and privacy is particularly difficult in high-pressure emergency settings. At the same time, healthcare legislation clearly stipulates that patients must be treated with respect and that their dignity should be safeguarded. Besides the challenges inherent in high-pressure emergency settings, this may also be reinforced by the fact that existential and relational aspects of care are seldom emphasized in clinical guidelines. An analysis of Swedish emergency care guidelines for frail older adults—conducted as part of a national survey study—showed that relational care needs were not addressed (0%), whereas physical care needs were emphasized (91%). This suggests that important dimensions of holistic care were overlooked [35]. Protecting patients’ dignity and privacy under these conditions requires more than physical restructuring; it calls for a cultural shift that prioritizes these principles. Small acts—screening a patient off, speaking softly, or offering clear explanations—might mitigate their existential exposure and restore a sense of personhood.

While person-centered care is widely recognized as essential for high-quality healthcare [14], our findings highlight the considerable challenges in achieving it in the emergency room. Time pressure, task orientation, and organizational routines frequently constrain opportunities for genuine connection and personalized care. In many cases, care is organized around the needs and competencies of the organization rather than the patient, resulting in service-oriented rather than need-oriented delivery [36] and leaving existential concerns insufficiently addressed. In the Swedish context, structural and organizational factors such as variation in staffing ratios, the absence of formal training requirements for acute care RNs, and traditional divisions of responsibility further reinforce a focus on rapid medical stabilization [37]. These systemic conditions shape care delivery beyond individual competence or intention. Importantly, even within these constraints, our results indicate that brief, attuned interactions—such as eye contact, verbal reassurance, or small gestures of presence—can have profound significance for patients, supporting a sense of safety and meaning. While the Fundamentals of Care framework [12] emphasizes the integration of physical, relational, and psychosocial dimensions, existential needs are not explicitly articulated within the elements described under the psychosocial dimension in its current version. As noted above, existential aspects were considered during the development of the framework but were not retained in its current version following the Delphi study [12], not due to lack of importance but rather due to lack of consensus regarding terminology. Our findings suggest that existential needs extend beyond the existing terminology of the framework and therefore warrant explicit conceptual recognition. For example, patients described withholding existential concerns due to perceived lack of time and being involuntarily exposed to others’ suffering and death—experiences that extend beyond existing concepts such as dignity, presence, or information. Since the original studies from which this secondary analysis derives were conducted with the Fundamentals of Care framework as an underpinning, the present results can be understood as extending its scope. By making existential needs more visible, this study might contribute to the Fundamentals of Care framework [12], pointing to the value of explicitly incorporating existential needs as a fundamental element of person-centered care.

Ultimately, improving emergency care requires more than individual effort—it demands organizational commitment to ethical practice, professional development, and cultural change. Recognizing existential needs as legitimate and actionable within emergency settings is not only ethically necessary but also foundational to high-quality, person-centered fundamental care.

### Study strengths and limitations

The strength of this study lies in its engagement with diverse data sources, which enabled a rich and nuanced understanding of existential needs from multiple perspectives. As the interpretation was supported by concepts identified as relevant to nursing and emergency care [19], it helped illuminate how existential needs were expressed without limiting the exploratory nature of the analysis.

However, this work has limitations that should be considered when evaluating the overall findings. The broad, overlapping nature of existential concepts such as suffering, pain, and crisis posed challenges in delineating distinct themes. The use of pre-existing data, while rich, means that the original data collection was not tailored specifically to the current research questions; while this allowed for authentic expressions of existential concern, it may also have limited the depth of exploration in some areas. Additionally, the study was conducted with data from a Swedish emergency room context, which may influence the transferability of findings to other cultural or healthcare settings. Additionally, the absence of participant validation (e.g. member checking) may limit the confirmability of the findings.

### Recommendations for further research

Based on this study’s findings, future research could involve observational studies in the emergency room, focusing on expressions of existential needs and associated ethical challenges. Such studies could offer real-time insight into how healthcare professionals navigate these needs within organizational constraints, communication, and presence. Future studies could also explore existential needs at the organizational level, complementing individual and relational perspectives. This would complement interview-based findings and deepen the understanding of both patient experience and the delivery of person-centered care in acute settings. Future research and practice should focus on interventions for integrating existential support into emergency care routines, fostering person-centered environments, and supporting healthcare professionals in navigating the ethical complexities of acute care.

### Implications for policy and practice

Existential support should be recognized as a vital component in the emergency room context. Training and organizational culture should enable healthcare professionals to identify and respond to patients’ existential needs even in acute conditions. Organizational strategies that protect dignity and privacy, and foster respectful communication, can reduce patients’ sense of exposure and alienation. Integrating person-centered fundamental care into emergency practice requires acknowledging the patient as a whole person, beyond their clinical condition, and making room for relational and psychosocial support alongside medical interventions. Providing structured opportunities for ethical reflection and collegial dialogue might help RNs navigate the moral tensions between life-saving procedures and holistic care. Embedding these principles into education, policy, and clinical guidelines might reinforce professional integrity and strengthen the delivery of person-centered fundamental care in emergency settings.

## Conclusion

This study indicates that existential needs—such as fear, vulnerability, and the search for meaning—are present yet overlooked in the emergency room, where medical urgency and organizational routines dominate. Furthermore, existential needs are not only present but deeply intertwined with the realities of receiving and providing care in the emergency room. Patients face sudden transitions, proximity to death, and emotional isolation, while RNs navigate organizational constraints in their effort to provide nursing care. The emergency room, designed primarily for medical stabilization, thus becomes a place where questions of meaning and mortality inevitably emerge. Existential expressions are conveyed through both verbal and non-verbal means, and are often mediated by the environment, communication, and presence. Even brief, genuine interactions can help address existential distress and preserve dignity, yet privacy and ethical tensions remain fragile. Recognizing and responding to these needs—integrated with rather than separate from the medical care—should be considered an essential component of person-centered fundamental care.

## Data Availability

Data generated during and/or analyzed during the study are not publicly available due to ethical restrictions and privacy.

## Abbreviations

ED: Emergency Department
RN: Registered Nurse
